# The Variance-Gamma Product Distribution

**DOI:** 10.1007/s00025-025-02499-y

**Published:** 2025-09-23

**Authors:** Robert E. Gaunt, Siqi Li, Heather L. Sutcliffe

**Affiliations:** Department of Mathematics, The University of Manchester, Oxford Road, M13 9PL Manchester, UK

**Keywords:** Variance-gamma distribution, Product distribution, Laplace distribution, Normal distribution, Meijer *G*-function, Primary 60E05, 62E15, Secondary 33C60, 41A60

## Abstract

We derive the exact probability density function of the product of *N* independent variance-gamma random variables with zero location parameter. We then apply this formula to derive formulas for the cumulative distribution function and characteristic function, as well as asymptotic approximations for the density, tail probabilities and quantile function. From our general results, we deduce closed-form formulas for the density, cumulative distribution function and characteristic function of the product of *N* independent asymmetric Laplace random variables and mixed products of independent Laplace and centred normal random variables.

## Introduction

The variance-gamma (VG) distribution with parameters $m > -1/2$, $0\le |\beta |<\alpha$, $\mu \in \mathbb {R}$, which we denote by $\textrm{VG}(m,\alpha ,\beta ,\mu )$, has probability density function (PDF)

1.1 $$\begin{aligned} f(x) = \frac{\gamma ^{2m+1}}{\sqrt{\pi }(2\alpha )^m \Gamma (m+1/2)} \textrm{e}^{\beta (x-\mu )}|x-\mu |^{m} K_{m}(\alpha |x-\mu |), \quad x\in \mathbb {R}, \end{aligned}$$

where $\gamma ^2=\alpha ^2-\beta ^2$ and $K_{m} (x) =\int _0^\infty \textrm{e}^{-x\cosh (t)}\cosh (m t)\,\textrm{d}t$ is the modified Bessel function of the second kind. The VG distribution is also referred to as the Bessel function distribution [1], the generalized Laplace distribution [2, Section 4.1] and the McKay Type II distribution [3]. Other parametrisations can also be found in [2, 4, 5]. Following the seminal works [5, 6], the VG distribution has been widely used in financial modelling; further application areas are given in the review [7] and Chapter 4 of the book [2]. In this paper, we set $\mu =0$. On further setting $\beta =0$, the PDF (1.1) is symmetric about the origin, and in this paper we will refer to the $\textrm{VG}(m,\alpha ,0,0)$ distribution as the *symmetric variance-gamma* distribution.

Let $X_1,\ldots ,X_N$ be independent VG random variables with $X_i\sim \textrm{VG}(m_i,\alpha _i,\beta _i,0)$, $m_i>-1/2$, $0\le |\beta _i|<\alpha _i$, $i=1,\ldots ,N$. In this paper, we obtain an exact formula for the PDF of the product $Z=\prod _{i=1}^N X_i$, which is expressed as an infinite series in terms of the Meijer *G*-function. Our formula generalises one of [8] for the product of two independent VG random variables (the $N=2$ case) and one of [9] for the PDF of the product *Z* in the case $\beta _1=\cdots =\beta _N=0$ (a product of symmetric VG random variables). We derive our formula using the theory of Mellin transforms, a method which has been used to find exact formulas for the PDFs of products of many important continuous random variables; see, for example, [10–16].

In the symmetric case $\beta _1=\cdots =\beta _N=0$, the PDF of the product *Z* reduces to a single Meijer *G*-function, whilst when $m_1,\ldots ,m_N$ are half-integers the PDF of *Z* can be represented as a finite summation of Meijer *G*-functions. In these more tractable cases, we apply our formulas for the PDF to deduce closed-form formulas for the cumulative distribution function (CDF) and characteristic function. Returning to the general case $m_i>-1/2$, $0\le |\beta _i|<\alpha _i$, $i=1,\ldots ,N$, we also derive asymptotic approximations for the PDF, tail probabilities and the quantile function. We state and prove these results in Section 2.

The $\textrm{VG}(1/2,\alpha ,\beta ,0)$ distribution corresponds to the asymmetric Laplace distribution (with zero location parameter) and the classical symmetric Laplace distribution is obtained from further setting $\beta =0$. Thus from our general results of Section 2 we obtain formulas for the PDF, CDF and characteristic function of the product of *N* independent asymmetric Laplace random variables. These formulas (which are given in Section 3.1) fill a surprising gap in the literature, as such formulas were previously only available in [8, 17] for the $N=2$ case. The product of two independent zero mean normal random variables is also VG distributed [4], and in Section 3.2 we deduce new results for the distribution of mixed products of independent Laplace and zero mean normal random variables. More generally, the product of two correlated zero mean normal random variables is VG distributed [18], and so we are also able to deduce an exact formula for the PDF of the product of 2*N* jointly correlated zero mean normal random variables with a block diagonal covariance matrix (see Section 3.3).

*Notation.* We let $S_N$ denote the power set of $\{1,\ldots ,N\}$, that is the set of all subsets of $\{1,\ldots ,N\}$. We let $\sigma \in S_N$ and define its complement $\sigma ^{c}=\{1,\ldots ,N\}\setminus \sigma$. We further let $S_N^+=\{\sigma \in S_N:\,|\sigma | \text { is even }\}$ and $S_N^-=\{\sigma \in S_N:\,|\sigma | \text { is odd }\}$. Also, $\textrm{sgn}(x)$ denotes the sign function, $\textrm{sgn}(x)=-1$ for $x<0$, $\textrm{sgn}(0)=0$, $\textrm{sgn}(x)=1$ for $x>0$. Lastly, we use the convention that the empty product is set to 1.

In order to simplify formulae, we let $\eta = 1/\prod ^N_{i=1} \Gamma (m_i+1/2)$ and $\xi =\prod ^N_{i=1} \alpha _i$. We also let $\gamma _i^2 = \alpha _i^2-\beta _i^2$ and $\lambda _i^{\pm }=\alpha _i\pm \beta _i$, $i=1,\ldots ,N$. Finally, for $\sigma \in S_N$, we let $\omega _\sigma =\prod _{k \in \sigma }\prod _{l \in \sigma ^c}(\alpha _k+\beta _k)(\alpha _l-\beta _l)$. In the case $(\alpha _i,\beta _i)=(\alpha ,\beta )$ for $i=1,\ldots ,N$, we have $\omega _\sigma =(\alpha +\beta )^{|\sigma |}(\alpha -\beta )^{N-|\sigma |}$.

## The VG Product Distribution

### Probability Density Function

We begin by obtaining an exact formula for the PDF of the product $Z = \prod _{i=1}^{N}X_i$. The formula is expressed as an infinite series given in terms of the Meijer *G*-function (defined in Appendix A).

#### Theorem 2.1

Let $X_1,\ldots ,X_N$ be *N* independent VG distributed random variables, where $X_i \sim \textrm{VG}(m_i,\alpha _i,\beta _i,0)$ with $m_i> -1/2$ and $0 \le |\beta _i| < \alpha _i$, for $i=1,\ldots ,N$. Let $Z = \prod _{i=1}^{N}X_i$. Then

2.1 $$\begin{aligned} f_Z(z)&= \frac{\eta }{2^{2N-1}\pi ^{N/2}}\bigg \{\prod _{i=1}^{N}\frac{\gamma _i^{2m_i+1}}{\alpha _i^{2m_i}}\bigg \}\nonumber \\  &\quad \times \sum ^\infty _{j_1=0} \ldots \sum ^\infty _{j_N=0} \frac{(2\beta _{1}/\alpha _1)^{j_1} }{ j_1! } \ldots \frac{(2\beta _N/\alpha _N)^{j_N}}{j_N!} a_{j_1,\ldots ,j_N}(z) \nonumber \\  &\quad \times G^{2N,0}_{0,2N}\bigg ( \frac{\xi ^{2}}{4^N}z^2\, \bigg |\, { - \atop \tfrac{j_1}{2},\ldots , \tfrac{j_N}{2}, m_1+\tfrac{j_1}{2}, \ldots , m_N+\tfrac{j_N}{2} } \bigg ),\quad z\in \mathbb {R}, \end{aligned}$$

where $a_{j_1,\ldots ,j_N}(z)=\sum _{\sigma \in S_N^+ } \prod _{k \in \sigma } (-1)^{j_k}$ for $z>0$ and $a_{j_1,\ldots ,j_N}(z)=\sum _{\sigma \in S_N^-} \prod _{k \in \sigma } (-1)^{j_k}$ for $z<0$.

#### Remark 2.2

In the case of a product of independent symmetric VG random variables ($\beta _1=\cdots =\beta _N=0$), the PDF $f_Z(z)$ is symmetric about the origin and reduces to a single *G*-function:

2.2 $$\begin{aligned} f_Z(z) = \frac{\xi \eta }{2^N \pi ^{N/2}} G^{2N,0}_{0,2N} \bigg ( \frac{\xi ^{2}}{4^N}z^2\, \bigg |\, \begin{aligned} - \atop 0, \ldots ,0, \, m_1,\ldots , m_N \end{aligned} \bigg ),\quad z\in \mathbb {R}, \end{aligned}$$

a formula that had earlier been derived by [9]. When $N-1$ of the skewness parameters are equal to zero, the PDF (2.1) simplifies to a single infinite series. Without loss of generality, set $\beta _2=\cdots =\beta _N=0$. Then, for $z\in \mathbb {R}$,

$$\begin{aligned} f_Z(z)&=\frac{\gamma _1^{2m_1+1}\alpha _2\cdots \alpha _N\eta }{2^{N}\pi ^{N/2}\alpha _1^{2m_1}} \sum _{k=0}^{\infty }\frac{1}{(2k)!}\bigg (\frac{2\beta _1}{\alpha _1}\bigg )^{2k}\\  &\quad \times G^{2N,0}_{0,2N}\bigg ( \frac{\xi ^2}{4^N}z^2\, \bigg |\, { - \atop k,0,\ldots ,0,m_1+k,m_2, \ldots , m_N } \bigg ), \end{aligned}$$

where we used that $a_{j,0,\ldots ,0}(z)=2^{N-2}(1+(-1)^{j})$. More generally, if $N-k$ of the skewness parameters are equal to zero then the PDF (2.1) reduces to a *k*-fold infinite series.

#### Proof

We begin by considering the case $z>0$. We will need the following notation. For a random variable *V*, we denote its positive and negative parts $V^+$ and $V^-$ by $V^+=\max \{V,0\}$ and $V^-=\max \{-V,0\}$. Recall that $Z=\prod _{i=1}^NX_i$, where $X_1,\ldots ,X_N$ are independent with $X_i \sim \textrm{VG}(m_i,\alpha _i,\beta _i,0)$, $i=1,\ldots ,N$. The Mellin transform of $Z^+$ is then given by

2.3 $$\begin{aligned} \mathcal {M}_{f_{Z^+}}(s)&= \sum _{\sigma \in S_N^+} \bigg \{\prod _{k \in \sigma } \prod _{l \in \sigma ^c }\mathcal {M}_{f_{X_k^-}}(s) \mathcal {M}_{f_{X_l^+}}(s)\bigg \}, \end{aligned}$$

where the Mellin transforms for $X_l^+$ and $X_k^-$, which were obtained by [8], are given by

2.4 $$\begin{aligned} \mathcal {M}_{f_{X_l^+}}(s)&=\frac{(\gamma _l/\alpha _l)^{2m_l+1}}{2\sqrt{\pi }\Gamma (m_l+1/2)}\sum _{j_l=0}^{\infty }\frac{(2\beta _l/\alpha _l)^{j_l}}{(j_l)!}\frac{2^s}{\alpha _l^s}\nonumber \\&\quad \times \Gamma \Big (\frac{s}{2}+\frac{j_l+1}{2}\Big )\Gamma \Big (\frac{s}{2}+m_l+\frac{j_l+1}{2}\Big ), \end{aligned}$$

2.5 $$\begin{aligned} \mathcal {M}_{f_{X_k^-}}(s)&=\frac{(\gamma _k/\alpha _k)^{2m_k+1}}{2\sqrt{\pi }\Gamma (m_k+1/2)}\sum _{j_k=0}^{\infty }\frac{(-2\beta _k/\alpha _k)^{j_k}}{(j_k)!}\frac{2^s}{\alpha _k^s}\nonumber \\  &\quad \times \Gamma \Big (\frac{s}{2}+\frac{j_k+1}{2}\Big ) \Gamma \Big (\frac{s}{2}+m_k+\frac{j_k+1}{2}\Big ). \end{aligned}$$

The PDF of $Z^+$ can be found on taking the inverse Mellin transform of (2.3), and so we calculate

2.6 $$\begin{aligned}&\mathcal {M}^{-1} \bigg ( \frac{(2^N)^s}{\xi ^s} \prod ^N_{i=1} \Gamma \Big (\frac{s}{2}+\frac{j_i+1}{2}\Big ) \Gamma \Big (\frac{s}{2}+m_i+\frac{j_i+1}{2}\Big ) \bigg ) \nonumber \\&\quad = \frac{1}{2 \pi \textrm{i}} \lim _{U \rightarrow \infty } \int ^{c+\textrm{i}U}_{c-\textrm{i}U} z^{-s-1} \frac{(2^N)^{s}}{\xi ^s } \prod ^N_{i=1} \Gamma \Big (\frac{s}{2}+\frac{j_i+1}{2}\Big ) \Gamma \Big (\frac{s}{2}+m_i+\frac{j_i+1}{2}\Big )\, \textrm{d}s \nonumber \\&\quad = \frac{\xi }{2^{N-1}} G^{2N,0}_{0,2N} \bigg ( \frac{\xi ^2}{4^N}z^2\, \bigg |\, \begin{aligned} -\atop \tfrac{j_1}{2},\ldots , \tfrac{j_N}{2}, m_1+\tfrac{j_1}{2}, \ldots , m_N+\tfrac{j_N}{2} \end{aligned} \bigg ), \end{aligned}$$

where $c > 0$. Here we calculated the contour integral by using the contour integral representation (A.1) of the *G*-function. The desired formula for the PDF of *Z* for $z>0$ now follows from (2.3), (2.4), (2.5) and (2.6). For $z<0$, we use that the Mellin transform of $Z^-$ is given by

$$\begin{aligned} \mathcal {M}_{f_{Z^-}}(s)=\sum _{\sigma \in S_N^-}\bigg \{ \prod _{k \in \sigma } \prod _{l \in \sigma ^c} \mathcal {M}_{f_{X_k^-}}(s)\mathcal {M}_{f_{X_l^+}}(s)\bigg \}, \end{aligned}$$

and by a similar argument we obtain the desired formula (2.1) for the PDF of *Z* for $z<0$. $\square$

We now provide a simplified formula for the PDF of $Z=\prod _{i=1}^{N}X_i$ in the special case that $m_1,\ldots ,m_N$ are half-integers. In this case, the PDF can be expressed as an *N*-fold finite sum rather than an *N*-fold infinite series. The Meijer *G*-functions in the series are also of lower order.

#### Proposition 2.3

Suppose that $m_1-1/2,\ldots ,m_N-1/2\ge 0$ are non-negative integers. Then, for $z\in \mathbb {R}$,

2.7 $$\begin{aligned} f_Z(z)&=\bigg \{\prod _{i=1}^{N}\frac{\gamma _i^{2m_i+1}}{(2\alpha _i)^{m_i+1/2}(m_i-1/2)!}\bigg \}\sum _{j_1=0}^{m_1-1/2}\cdots \sum _{j_N=0}^{m_N-1/2}\frac{(m_1-1/2+j_1)!}{(m_1-1/2-j_1)!j_1!}\nonumber \\&\quad \times \cdots \frac{(m_N-1/2+j_N)!}{(m_N-1/2-j_N)!j_N!}\sum _{\sigma \in A(z)}\bigg \{\prod _{k \in \sigma }\prod _{l \in \sigma ^c}\frac{(\alpha _k+\beta _k)^{j_k+1/2-m_k}}{(2\alpha _k)^{j_k}}\nonumber \\&\quad \times \frac{(\alpha _l-\beta _l)^{j_l+1/2-m_l}}{(2\alpha _l)^{j_l}}\bigg \} G^{N,0}_{0,N} \bigg ( \omega _\sigma |z|\, \bigg |\, \begin{aligned} -\atop m_1-\tfrac{1}{2}-j_1, \ldots , m_N-\tfrac{1}{2}-j_N \end{aligned} \bigg ),\nonumber \\ \end{aligned}$$

where $A(z)=S_N^+$ for $z>0$ and $A(z)=S_N^-$ for $z<0$.

#### Remark 2.4

When $\beta _1=\cdots =\beta _N=0$ the PDF (2.7) simplifies to the following. For $z\in \mathbb {R}$,

2.8 $$\begin{aligned} f_Z(z)&=\frac{\xi }{2}\bigg \{\prod _{i=1}^{N}\frac{2^{1/2-m_i}}{(m_i-1/2)!}\bigg \}\sum _{j_1=0}^{m_1-1/2}\cdots \sum _{j_N=0}^{m_N-1/2}\frac{(m_1-1/2+j_1)!}{(m_1-1/2-j_1)!j_1!2^{j_1}}\times \cdots \nonumber \\  &\quad \times \frac{(m_N-1/2+j_N)!}{(m_N-1/2-j_N)!j_N!2^{j_N}}\nonumber \\  &\quad \times G^{N,0}_{0,N}\bigg ( \xi |z|\,\bigg |\, \begin{aligned} -\atop m_1-\tfrac{1}{2}-j_1, \ldots , m_N-\tfrac{1}{2}-j_N \end{aligned} \bigg ). \end{aligned}$$

#### Proof

We first consider the case $z>0$. We begin by computing the Mellin transform of $X_i^+$:

$$\begin{aligned} \mathcal {M}f_{X_i^+}(s)&=\int _{0}^{\infty }x^s\frac{\gamma _i^{2m_i+1}}{\sqrt{\pi }(2\alpha _i)^{m_i}(m_i-1/2)!}\textrm{e}^{\beta _ix}x^{m_i}K_{m_i}(\alpha _ix)\,\textrm{d}x\\&=\frac{\gamma _i^{2m_i+1}}{(2\alpha _i)^{m_i+1/2}(m_i-1/2)!}\sum _{j_i=0}^{m_i-1/2}\frac{(m_i-1/2+j_i)!}{(m_i-1/2-j_i)!j_i!}(2\alpha _i)^{-j_i}\\&\quad \times \int _{0}^{\infty }\textrm{e}^{-(\alpha _i-\beta _i)x}x^{m_i-1/2-j_i+s}\,\textrm{d}x\\&=\frac{\gamma _i^{2m_i+1}}{(2\alpha _i)^{m_i+1/2}(m_i-1/2)!}\sum _{j_i=0}^{m_i-1/2}\frac{(m_i-1/2+j_i)!}{(m_i-1/2-j_i)!j_i!}(2\alpha _i)^{-j_i}\\&\quad \times (\alpha _i-\beta _i)^{j_i-m_i-1/2}\frac{\Gamma (m_i+s+1/2-j_i)}{(\alpha _i-\beta _i)^s}, \end{aligned}$$

where in the second step we used the elementary representation of the modified Bessel function of the second kind ${K} _{m+1/2}(x)=\sqrt{\pi /(2x)}\sum _{j=0}^m\frac{(m+j)!}{(m-j)!j!}(2x)^{-j}\textrm{e}^{-x}$ for $m=0,1,2,\ldots$, and in the final step we used the well-known integral representation of the gamma function to evaluate the integral. Similarly, for $X_i^-$ we have

$$\begin{aligned} \mathcal {M}f_{X_i^-}(s)&=\frac{\gamma _i^{2m_i+1}}{(2\alpha _i)^{m_i+1/2}(m_i-1/2)!}\sum _{j_i=0}^{m_i-1/2}\frac{(m_i-1/2+j_i)!}{(m_i-1/2-j_i)!j_i!}(2\alpha _i)^{-j_i}\\&\quad \times (\alpha _i+\beta _i)^{j_i-m_i-1/2}\frac{\Gamma (m_i+s+1/2-j_i)}{(\alpha _i+\beta _i)^s}. \end{aligned}$$

The Mellin transform of $Z^+$ is given by

2.9 $$\begin{aligned}&\mathcal {M}f_{Z^+}(s)\nonumber \\  &=\sum _{\sigma \in S_N^+}\bigg \{\prod _{k \in \sigma }\prod _{l \in \sigma ^c}\mathcal {M}_{f_{X_k^-}}(s)\mathcal {M}_{f_{X_l^+}}(s)\bigg \}\nonumber \\  &=\bigg \{\prod _{i=1}^{N}\frac{\gamma _i^{2m_i+1}}{(2\alpha _i)^{m_i+1/2}(m_i-1/2)!}\bigg \}\nonumber \\  &\quad \times \sum _{j_1=0}^{m_1-1/2}\cdots \sum _{j_N=0}^{m_N-1/2}\frac{(m_1-1/2+j_1)!}{(m_1-1/2-j_1)!j_1!}\cdots \frac{(m_N-1/2+j_N)!}{(m_N-1/2-j_N)!j_N!}\nonumber \\  &\quad \times \sum _{\sigma \in S_N^+}\bigg \{\prod _{k \in \sigma }\prod _{l \in \sigma ^c}\frac{(\alpha _k+\beta _k)^{j_k-1/2-m_k}}{(2\alpha _k)^{j_k}}\cdot \frac{(\alpha _l-\beta _l)^{j_l-1/2-m_l}}{(2\alpha _l)^{j_l}}\nonumber \\  &\quad \times \frac{\Gamma (m_k+s+1/2-j_k)}{(\alpha _k-\beta _k)^{s}}\frac{\Gamma (m_l+s+1/2-j_l)}{(\alpha _l+\beta _l)^{s}}\bigg \}. \end{aligned}$$

Taking the inverse Mellin transform of (2.9) yields the PDF of $Z^+$ and we are left to find

$$\begin{aligned}&\mathcal {M}^{-1}\bigg (\prod _{k \in \sigma }\prod _{l \in \sigma ^c }\frac{\Gamma (m_k+s+1/2-j_k)}{(\alpha _k+\beta _k)^s}\frac{\Gamma (m_l+s+1/2-j_l)}{(\alpha _l-\beta _l)^s}\bigg )\\  &\quad =\mathcal {M}^{-1}\bigg (\bigg \{\prod _{i=1}^{N}\Gamma (m_i+s+1/2-j_i)\bigg \} \bigg \{\prod _{k \in \sigma }\prod _{l \in \sigma ^c}(\alpha _k+\beta _k)^{-s}(\alpha _l-\beta _l)^{-s}\bigg \}\bigg )\\  &\quad =\frac{1}{2\pi \text {i}}\lim _{U \rightarrow \infty }\int _{c+\text {i}U}^{c-\text {i}U}z^{-s-1}\bigg \{\prod _{i=1}^{N}\Gamma (m_i+s+1/2-j_i)\bigg \}\\  &\qquad \times \bigg \{\prod _{k\in \sigma }\prod _{l \in \sigma ^c}(\alpha _k+\beta _k)^{-s}(\alpha _l-\beta _l)^{-s}\bigg \}\,\text {d}s\\  &\quad =\bigg \{\prod _{k\in \sigma }\prod _{l \in \sigma ^c}(\alpha _k+\beta _k)(\alpha _l-\beta _l)\bigg \} \\  &\qquad \times G^{N,0}_{0,N}\bigg ( z\prod _{k \in \sigma }\prod _{l \in \sigma ^c}(\alpha _k+\beta _k)(\alpha _l-\beta _l)\,\bigg | \,\begin{aligned} -\atop m_1-\tfrac{1}{2}-j_1, \ldots , m_N-\tfrac{1}{2}-j_N \end{aligned}\bigg ), \end{aligned}$$

where $c>0$ and in the final step we evaluated the contour integral by using a change of variables and the contour integral representation (A.1) of the Meijer *G*-function. We now have

$$\begin{aligned} f_{Z^+}(z)&=\bigg \{\prod _{i=1}^{N}\frac{\gamma _i^{2m_i+1}}{(2\alpha _i)^{m_i+1/2}(m_i-1/2)!}\bigg \}\\  &\quad \times \!\sum _{j_1=0}^{m_1-1/2}\!\cdots \! \sum _{j_N=0}^{m_N-1/2}\! \frac{(m_1-1/2+j_1)!}{(m_1-1/2-j_1)!j_1!}\cdots \frac{(m_N-1/2+j_N)!}{(m_N-1/2-j_N)!j_N!}\\  &\quad \times \sum _{\sigma \in S_N^+}\bigg \{\prod _{k \in \sigma }\prod _{l \in \sigma ^c}\frac{(\alpha _k+\beta _k)^{j_k+1/2-m_k}}{(2\alpha _k)^{j_k}}\cdot \frac{(\alpha _l-\beta _l)^{j_l+1/2-m_l}}{(2\alpha _l)^{j_l}}\bigg \}\\  &\quad \times G^{N,0}_{0,N} \bigg (\omega _\sigma z \,\bigg |\, \begin{aligned} -\atop m_1-\tfrac{1}{2}-j_1, \ldots , m_N-\tfrac{1}{2}-j_N \end{aligned}\bigg ). \end{aligned}$$

By a similar argument we can obtain the desired formula for the PDF of *Z* for $z<0$. $\square$

### Cumulative Distribution Function

Denote the CDF of $Z = \prod ^N_{i=1} X_i$ by $F_Z(z) = \mathbb {P}(Z \le z)$. We now obtain an exact formula for the CDF of the product of *N* independent symmetric VG random variables.

#### Theorem 2.5

Suppose that $\beta _1=\cdots =\beta _N=0$ and $m_1,\ldots ,m_N>-1/2$, $\alpha _1,\ldots ,\alpha _N>0$. Then, for $z \in \mathbb {R}$,

2.10 $$\begin{aligned} F_Z(z)&=\frac{1}{2}+ \frac{\xi \eta z }{ 2^{N+1} \pi ^{N/2} } G^{2N,1}_{1,2N+1}\bigg ( \frac{\xi ^2}{4^{N}}z^2 \,\bigg |\,{ \frac{1}{2} \atop 0,\ldots ,0,m_1,\ldots ,m_N,-\frac{1}{2}}\bigg )\\&=\frac{1}{2}+ \frac{\eta \,\textrm{sgn}(z)}{ 2 \pi ^{N/2} } G^{2N,1}_{1,2N+1}\bigg ( \frac{\xi ^2}{4^{N}}z^2\, \bigg |\,{ 1 \atop \frac{1}{2},\ldots ,\frac{1}{2},m_1+\frac{1}{2},\ldots ,m_N+\frac{1}{2},{0}}\bigg ). \end{aligned}$$

#### Proof

We only consider the case $z>0$, since the PDF (2.2) is symmetric about the origin when $\beta _1=\cdots =\beta _N=0$. Now, for $z>0$,

2.11 $$\begin{aligned} F_Z(z)&=\frac{1}{2}+ \frac{\xi \eta }{ 2^N \pi ^{N/2} } \int _0^z G^{2N,0}_{0,2N}\bigg ( \frac{\xi ^2 }{2^{2N}} y^2\, \bigg |\,{- \atop 0,\ldots ,0,m_1,\ldots ,m_N}\bigg )\,\textrm{d}y \nonumber \\&=\frac{1}{2}+ \frac{\eta }{ 2\pi ^{N/2} } \int _0^{\tfrac{\xi ^2z^2}{2^{2N}}}u^{-1/2} G^{2N,0}_{0,2N}\bigg ( u \, \bigg |\,{- \atop 0,\ldots ,0,m_1,\ldots ,m_N}\bigg )\,\textrm{d}u \nonumber \\&=\frac{1}{2}+\frac{\eta }{2\pi ^{N/2}}\bigg [u^{1/2}G^{2N,1}_{1,2N+1}\bigg ( u \, \bigg |\,{\tfrac{1}{2} \atop 0,\ldots ,0,m_1,\ldots ,m_N, -\tfrac{1}{2}}\bigg )\bigg ]^{\tfrac{\xi ^2z^2}{2^{2N}}}_{0} \nonumber \\&= \frac{1}{2}+ \frac{\xi \eta z }{ 2^{N+1} \pi ^{N/2} } G^{2N,1}_{1,2N+1}\bigg ( \frac{\xi ^2}{4^{N}}z^2\, \bigg |\,{ \frac{1}{2} \atop 0,\ldots ,0,m_1,\ldots ,m_N,-\frac{1}{2}}\bigg ). \end{aligned}$$

Here we evaluated the integral using (A.6) and in the last step used that the *G*-function in (2.12) evaluated at $u = 0$ is equal to zero, which follows easily from the contour integral definition (A.1) of the Meijer *G*-function. Lastly, (2.11) follows from (2.10) due to the relation (A.3). $\square$

When $m_1,\ldots ,m_N$ are half-integers, the CDF of *Z* can be expressed as an *N*-fold finite sum involving Meijer *G*-functions of lower order.

#### Proposition 2.6

Suppose that $m_1-1/2,\ldots ,m_N-1/2\ge 0$ are non-negative integers. Also, let $\beta _1=\cdots =\beta _N=0$ and $\alpha _1,\ldots ,\alpha _N>0$. Then, for $z \in \mathbb {R}$,

2.12 $$\begin{aligned} F_Z(z)= &   \frac{1}{2}+\frac{\textrm{sgn}(z)}{2}\bigg \{\prod _{i=1}^{N}\frac{2^{1/2-m_i}}{(m_i-1/2)!}\bigg \}\sum _{j_1=0}^{m_1-1/2}\cdots \nonumber \\  &   \times \sum _{j_N=0}^{m_N-1/2} \frac{(m_1-1/2+j_1)!}{(m_1-1/2-j_1)!j_1!2^{j_1}} \cdots \frac{(m_N-1/2+j_N)!}{(m_N-1/2-j_N)!j_N!2^{j_N}}\nonumber \\  &   \times \, G^{N,1}_{1,N+1} \bigg ( \xi |z|\,\bigg |\, \begin{aligned}&1 \\ m_1+\tfrac{1}{2}-j_1, \ldots&, m_N+\tfrac{1}{2}-j_N,0 \end{aligned} \bigg ). \end{aligned}$$

#### Proof

Since $\beta _1=\cdots =\beta _N=0$, the PDF of *Z*, as given by (2.8), is symmetric about the origin, and therefore $F_Z(z)=1/2+\textrm{sgn}(z)\int _{0}^{|z|}f_Z(y)\,\textrm{d}y$. We then have, for $z\in \mathbb {R}$,

2.13 $$\begin{aligned} F_Z(z)  &   =\frac{1}{2}+\frac{\text {sgn}(z)}{2}\bigg \{\prod _{i=1}^N\frac{2^{1/2-m_i}}{(m_i-1/2)!}\bigg \}\nonumber \\  &   \quad \times \sum _{j_1=0}^{m_1-1/2}\cdots \sum _{j_N=0}^{m_N-1/2} \frac{(m_1-1/2+j_1)!}{(m_1-1/2-j_1)!j_1!2^{j_1}}\times \cdots \nonumber \\  &   \quad \times \frac{(m_N-1/2+j_N)!}{(m_N-1/2-j_N)!j_N!2^{j_N}}\nonumber \\  &   \quad \times \int _{0}^{\xi |z|}G_{0,N}^{N,0}\bigg (u \; \bigg | \;{-\atop m_1-\frac{1}{2}-j_1,\ldots ,m_N-\frac{1}{2}-j_N}\bigg )\,\text {d}u\nonumber \\  &   =\frac{1}{2}+\frac{\text {sgn}(z)}{2}\bigg \{\prod _{i=1}^{N}\frac{2^{1/2-m_i}}{(m_i-1/2)!}\bigg \}\nonumber \\  &   \quad \times \sum _{j_1=0}^{m_1-1/2}\cdots \sum _{j_N=0}^{m_N-1/2}\frac{(m_1-1/2+j_1)!}{(m_1-1/2-j_1)!j_1!2^{j_1}}\times \cdots \nonumber \\  &   \quad \times \frac{(m_N-1/2+j_N)!}{(m_N-1/2-j_N)!j_N!2^{j_N}}\nonumber \\  &   \quad \times \bigg [G_{1,N+1}^{N,1}\bigg (u \; \bigg | \;{1\atop m_1+\frac{1}{2}-j_1,\ldots ,m_N+\frac{1}{2}-j_N,0}\bigg )\bigg ]^{\xi |z|}_0\nonumber \\  &   =\frac{1}{2}+\frac{\text {sgn}(z)}{2}\bigg \{\prod _{i=1}^{N}\frac{2^{1/2-m_i}}{(m_i-1/2)!}\bigg \}\nonumber \\  &   \quad \times \sum _{j_1=0}^{m_1-1/2}\cdots \sum _{j_N=0}^{m_N-1/2} \frac{(m_1-1/2+j_1)!}{(m_1-1/2-j_1)!j_1!2^{j_1}}\times \cdots \nonumber \\  &   \quad \times \frac{(m_N-1/2+j_N)!}{(m_N-1/2-j_N)!j_N!2^{j_N}}\nonumber \\  &   \quad \times G_{1,N+1}^{N,1}\bigg (\xi |z| \; \bigg | \;{1\atop m_1+\frac{1}{2}-j_1,\ldots ,m_N+\frac{1}{2}-j_N,0}\bigg ), \end{aligned}$$

where in the third step we evaluated the integral using (A.7). The final step follows from the fact that the *G*-function in (2.14) evaluated at $u=0$ is equal to zero, which can be readily seen from the contour integral representation (A.1) of the Meijer *G*-function. $\square$

In the following proposition, we provide a closed-form formula for the probability $\mathbb {P}(Z\le 0)$, expressed in terms of the Gaussian hypergeometric function (see [19, Chapter 15] for a definition). We make use of the following formula of [20]. Let $m>-1/2$, $0\le |\beta |<\alpha$. Then, for $X\sim \textrm{VG}(m,\alpha ,\beta ,0)$,

$$\begin{aligned} \mathbb {P}(X\le 0)&=P_{m,\alpha ,\beta }\\&:=\frac{1}{2}-\frac{(\beta /\alpha )\Gamma (m+1)}{\sqrt{\pi }\Gamma (m+1/2)}\bigg (1-\frac{\beta ^2}{\alpha ^2}\bigg )^{m+1/2} {_2F_1}\bigg (1,m+1;\frac{3}{2};\frac{\beta ^2}{\alpha ^2}\bigg ). \end{aligned}$$

#### Proposition 2.7

Let $$m_1,\ldots ,m_N>-1/2$$, $$0\le |\beta _i|<\alpha _i$$, $$i=1,\ldots ,N$$. Then

2.14 $$\begin{aligned} \mathbb {P}(Z\le 0)=\sum _{i=1}^{N}P_{m_i,\alpha _i,\beta _i}+\sum _{\begin{array}{c} \sigma \in S_N: \\ |\sigma |\ge 2 \end{array} }(-2)^{|\sigma |-1}\prod _{j \in \sigma }P_{m_j,\alpha _j,\beta _j}. \end{aligned}$$

In the case $(m_i,\alpha _i,\beta _i)=(m,\alpha ,\beta )$ for $i=1,\ldots ,N$, we have the simpler formula

2.15 $$\begin{aligned} \mathbb {P}(Z\le 0)=\frac{1}{2}-\frac{1}{2}(1-2P_{m,\alpha ,\beta })^N. \end{aligned}$$

#### Proof

A simple calculation shows that, for independent continuous random variables *U* and *V*,

2.16 $$\begin{aligned} \mathbb {P}(UV\le 0)=\mathbb {P}(U\le 0)+\mathbb {P}(V\le 0)-2\mathbb {P}(U\le 0)\mathbb {P}(V\le 0). \end{aligned}$$

Therefore in the $N=2$ case we have that $\mathbb {P}(Z\le 0)=P_{m_1,\alpha _1,\beta _1}+P_{m_2,\alpha _2,\beta _2}-2P_{m_1,\alpha _1,\beta _1}P_{m_2,\alpha _2,\beta _2}$. This confirms that equation (2.15) holds for $N=2$. The general case follows by a straightforward induction on *N* using equation (2.17). The proof of formula (2.16) in the case $(m_i,\alpha _i,\beta _i)=(m,\alpha ,\beta )$, $i=1,\ldots ,N$, follows from a similar inductive argument. $\square$

### Characteristic Function

In the following theorem, we obtain a closed-form formula for the characteristic function of the product of independent symmetric VG random variables. We write $\varphi _Z(t)=\mathbb {E}[\textrm{e}^{\textrm{i}tZ}]$ for the characteristic function of *Z*.

#### Theorem 2.8

Let $\beta _1=\cdots =\beta _N=0$ and $m_1,\ldots ,m_N>-1/2$, $\alpha _1,\ldots ,\alpha _N>0$. Then

2.17 $$\begin{aligned} \varphi _Z(t)&=\frac{\xi \eta }{2^{N-1}\pi ^{(N-1)/2}}|t|^{-1}G^{2N-1,1}_{1,2N-1} \bigg ( \frac{\xi ^2}{4^{N-1}t^2} \,\bigg |\, \begin{aligned} \tfrac{1}{2}\atop 0, \ldots ,0, \,m_1,\ldots , m_N \end{aligned} \bigg ),\quad t\in \mathbb {R}. \end{aligned}$$

#### Proof

As the PDF (2.2) of *Z* is an even function in *z*, $\varphi _Z(t)=\mathbb {E}[\cos (tZ)]$. We then get that

$$\begin{aligned} \varphi _Z(t)&=2\int _{0}^{\infty }\frac{\xi \eta }{2^N\pi ^{N/2}}\cos (tz)G^{2N,0}_{0,2N} \bigg ( \frac{\xi ^2}{4^N}z^2\, \bigg |\, \begin{aligned} -\atop 0, \ldots ,0, \,m_1,\ldots , m_N \end{aligned} \bigg )\,\textrm{d}z \\&=\frac{\xi \eta }{2^{N-1}\pi ^{N/2}}\frac{\sqrt{\pi }}{|t|}G^{2N,1}_{2,2N} \bigg ( \frac{\xi ^2}{4^{N-1}t^2}\, \bigg |\, \begin{aligned} {\tfrac{1}{2},0}\atop 0, \ldots ,0, \,m_1,\ldots , m_N \end{aligned} \bigg )\\&=\frac{\xi \eta }{2^{N-1}\pi ^{(N-1)/2}}|t|^{-1}G^{2N-1,1}_{1,2N-1} \bigg ( \frac{\xi ^2}{4^{N-1}t^2} \,\bigg |\, { \tfrac{1}{2}\atop 0, \ldots ,0, \,m_1,\ldots , m_N}\bigg ), \end{aligned}$$

where the integral was evaluated using equation 5.6.3(18) of [21] and the third equality was obtained by using (A.2). $\square$

When $m_1,\ldots ,m_N$ are half-integers, the characteristic function can be expressed as an *N*-fold finite sum involving Meijer *G*-functions of lower order, even for non-zero skewness parameters.

#### Proposition 2.9

Suppose that $m_1-1/2,\ldots ,m_N-1/2\ge 0$ are non-negative integers and that $0\le |\beta _i|<\alpha _i$, $i=1,\ldots ,N$. Then

2.18 $$\begin{aligned} \varphi _Z(t)&=\frac{\textrm{i}}{t}\bigg \{\prod _{i=1}^{N}\frac{\gamma _i^{2m_i+1}}{(2\alpha _i)^{m_i+1/2}(m_i-1/2)!}\bigg \}\nonumber \\  &\quad \times \sum _{j_1=0}^{m_1-1/2}\cdots \sum _{j_N=0}^{m_N-1/2} \frac{(m_1-1/2+j_1)!}{(m_1-1/2-j_1)!j_1!}\times \cdots \nonumber \\&\quad \times \frac{(m_N-1/2+j_N)!}{(m_N-1/2-j_N)!j_N!}\nonumber \\  &\quad \times \bigg \{\sum _{\sigma \in S_N^+}\bigg \{\prod _{k \in \sigma }\prod _{l \in \sigma ^c}\frac{(\alpha _k+\beta _k)^{j_k+1/2-m_k}}{(2\alpha _k)^{j_k}}\cdot \frac{(\alpha _l-\beta _l)^{j_l+1/2-m_l}}{(2\alpha _l)^{j_l}}\bigg \}\nonumber \\&\quad \times G^{N,1}_{1,N} \bigg (\frac{\textrm{i}\omega _\sigma }{t}\, \bigg |\, \begin{aligned}&0 \\ m_1-\tfrac{1}{2}-j_1, \ldots&,m_N-\tfrac{1}{2}-j_N \end{aligned} \bigg )\nonumber \\&\quad -\sum _{\sigma \in S_N^-}\bigg \{\prod _{k \in \sigma }\prod _{l \in \sigma ^c}\frac{(\alpha _k+\beta _k)^{j_k+1/2-m_k}}{(2\alpha _k)^{j_k}}\cdot \frac{(\alpha _l-\beta _l)^{j_l+1/2-m_l}}{(2\alpha _l)^{j_l}}\bigg \}\nonumber \\&\quad \times G^{N,1}_{1,N} \bigg (-\frac{\textrm{i}\omega _\sigma }{t}\, \bigg |\, \begin{aligned}&0 \\ m_1-\tfrac{1}{2}-j_1, \ldots&,m_N-\tfrac{1}{2}-j_N \end{aligned} \bigg )\bigg \},\quad t\in \mathbb {R}. \end{aligned}$$

#### Proof

Recall that under the conditions of the proposition a formula for the PDF of *Z* is given by (2.7). We then calculate $\varphi _Z(t)=\mathbb {E}[\textrm{e}^{\textrm{i}tZ}]=\int _{0}^{\infty }\textrm{e}^{\textrm{i}tz}f_Z(z)\,\textrm{d}z+\int _{-\infty }^{0}\textrm{e}^{\textrm{i}tz}f_Z(z)\,\textrm{d}z$, evaluating the integrals using formula 5.6.3(1) of [21]. $\square$

### Asymptotic Behaviour of the Distribution

Given that the PDF of the product *Z* for general parameter values takes a rather complicated form, it is of interest to study the asymptotic behaviour of the distribution. In this section, we derive asymptotic approximations for the PDF, tail probabilities and quantile function. We recall that we use the convention that the empty product is set to 1.

#### Asymptotic Behaviour of the Density at the Origin

##### Proposition 2.10

Let $$m_1,\ldots ,m_N>-1/2$$, $$0\le |\beta _i|<\alpha _i$$, $$i=1,\ldots ,N$$, where $$N\ge 2$$.

(i) Suppose that $m_1,\ldots ,m_N\ge 0$, and let $0 \le t \le N$ be the number of zeros among them. Without loss of generality, set $m_1= \cdots =m_t=0$ and $m_{t+1},\ldots , m_N > 0.$ Then, as $z \rightarrow 0,$
  2.19 $$\begin{aligned} f_Z(z) \sim \frac{2^{t-1} \gamma _1\cdots \gamma _t\gamma _{t+1}^{2m_{t+1}+1}\cdots \gamma _N^{2m_N+1} \eta }{ (N+t-1)! \pi ^{N/2} \alpha _{t+1}^{2m_{t+1}}\cdots \alpha _N^{2m_N} } \bigg \{ \prod ^{N-t}_{i=1} \Gamma (m_{t+i}) \bigg \} (- \ln |z|)^{N+t-1}. \end{aligned}$$
(ii) Suppose that $m_1 = \min \{m_1,\ldots ,m_N\} < 0.$ Without loss of generality, set $m_1= \cdots =m_t<m_{t+1},\ldots ,m_N$ for $1\le t\le N$. Then, as $z \rightarrow 0$,
  2.20 $$\begin{aligned} f_Z(z)&\sim \frac{\gamma _1^{2m_1+1}\cdots \gamma _N^{2m_N+1} (\Gamma (-m_1))^N\eta }{2^{ N (1+2m_1)} \pi ^{N/2} } \bigg ( \frac{\alpha _{t+1}^{2m_1}\cdots \alpha _N^{2m_1} }{\alpha _{t+1}^{2m_{t+1}}\cdots \alpha _N^{2m_N} } \bigg ) \nonumber \\&\quad \times \bigg \{\prod ^N_{i=t+1} \Gamma (m_i-m_1)\bigg \}\frac{(-2\ln |z|)^{t-1}}{(t-1)!}|z|^{2m_1}. \end{aligned}$$
(iii) When $N\ge 2$, the distribution of *Z* is unimodal with mode 0 for all parameter constellations.

##### Proof

Recall that the density function (2.1) for the product *Z* is an infinite summation of *G*-functions. Set $x=\xi z/2^N$. We will derive the limiting forms (2.20) and (2.21) by considering the asymptotic behaviour of the functions

$$\begin{aligned} g_{j_1,\ldots , j_N, m_1,\ldots ,m_N}(x)=G^{2N,0}_{0,2N} \bigg (x^2\, \bigg |\, \begin{aligned} -\atop \tfrac{j_1}{2},\ldots ,\tfrac{j_N}{2}, m_1+\tfrac{j_1}{2}, \ldots , m_N+\tfrac{j_N}{2} \end{aligned} \bigg ), \end{aligned}$$

in the limit $x\downarrow 0$, where $j_1,\ldots ,j_N\ge 0$. Note that since $g_{j_1,\ldots , j_N, m_1,\ldots ,m_N}(x)$ is an even function in *x* it does indeed suffice to consider the limit $x\downarrow 0$. We can express $g_{j_1,\ldots , j_N, m_1,\ldots ,m_N}(x)$ as a contour integral using (A.1):

2.21 $$\begin{aligned} g_{j_1,\ldots , j_N, m_1,\ldots ,m_N}(x)&=\frac{1}{2\pi \textrm{i}}\int _{L}\bigg \{\prod _{i=1}^{N}\Gamma \Big (s+\frac{j_i}{2}\Big )\Gamma \Big (s+m_i+\frac{j_i}{2}\Big )\bigg \}x^{-2s}\,\textrm{d}s \nonumber \\&=\sum _{s^*\in B}\textrm{Res}\bigg [\bigg \{\prod _{i=1}^{N}\Gamma \Big (s+\frac{j_i}{2}\Big )\Gamma \Big (s+m_i+\frac{j_i}{2}\Big )\bigg \}x^{-2s}, \,s=s^*\bigg ], \end{aligned}$$

where the contour *L* is a loop encircling the poles of the gamma functions and *B* is the set of these poles. We compute the residues in (2.22) for the two cases (i) and (ii) separately.

(i) Suppose $m_1,\ldots ,m_N\ge 0$ with $m_1=\cdots =m_t=0$ and $m_{t+1},\ldots ,m_N>0$. We will first consider the case $j_1=\cdots =j_N=0$. We calculate
  2.22 $$\begin{aligned} \textrm{Res}\bigg [(\Gamma (s))^{t+N}\bigg \{\prod _{i=t+1}^{N}\Gamma (s+m_i)\bigg \}x^{-2s},\, s=s^*\bigg ] \end{aligned}$$
  for all poles $s^*$ of the gamma functions in (2.23). We begin with the pole at $s=0$. On using the expansion $x^{-2s} =\exp (-2s\ln (x)) = \sum ^\infty _{i=0} (-2 \ln (x))^i s^i/i!$, and the asymptotic expansion $\Gamma (s)=1/s-\gamma +o(1)$ as $s\rightarrow 0$ ($\gamma$ is the Euler-Mascheroni constant), we obtain that, as $x\downarrow 0$,
  2.23 $$\begin{aligned}&\textrm{Res}\bigg [(\Gamma (s))^{t+N}\bigg \{\prod _{i=t+1}^{N}\Gamma (s+m_i)\bigg \}x^{-2s},\, s=0\bigg ]\nonumber \\&\quad \sim \bigg \{\prod _{i=t+1}^{N}\Gamma (m_{i})\bigg \}\frac{(-2\ln (x))^{N+t-1}}{(N+t-1)!}. \end{aligned}$$
  It is readily seen that for all other poles the residue (2.23) is of a smaller asymptotic order in the limit $x\downarrow 0$ than (2.24). It is also easily seen that for all other choices of $j_1,\ldots ,j_N\ge 0$, besides the case we considered of $j_1=\cdots =j_N=0$, that the residues in (2.22) are of a smaller asymptotic order in the limit $x\downarrow 0$ than (2.23). Thus, as $x\rightarrow 0$ (equivalently as $z\rightarrow 0$),
  2.24 $$\begin{aligned} g_{0,\ldots , 0, m_{t+1}, \ldots , m_N}(x)&\sim \bigg \{\prod _{i=t+1}^{N}\Gamma (m_{i})\bigg \}\frac{(-2\ln |x|)^{N+t-1}}{(N+t-1)!}\nonumber \\&\sim \bigg \{\prod _{i=t+1}^{N}\Gamma (m_{i})\bigg \}\frac{(-2\ln |z|)^{N+t-1}}{(N+t-1)!}. \end{aligned}$$
  Thus, since $g_{j_1,\ldots , j_N, m_1,\ldots ,m_N}(x)$ is of smaller asymptotic order in the limit $x\rightarrow 0$ (equivalently $z\rightarrow 0$) for all other choices of $j_1,\ldots ,j_N\ge 0$, on multiplying (2.25) through by the multiplicative constant of $g_{0,\ldots , 0, m_{t+1}, \ldots , m_N}(x)$ from the PDF (2.1) we obtain the limiting form (2.20).
(ii) Suppose $m_1=\min \{m_1,\ldots ,m_N\}<0$ and that $m_1=\cdots =m_t<m_{t+1},\ldots ,m_N$ for $1\le t\le N$. Since $m_1<0$, the dominant residue of (2.22) is now at the pole $s=-m_1$, and we have
  $$\begin{aligned}&\textrm{Res}\bigg [(\Gamma (s))^{N}(\Gamma (s+m_1))^t\bigg \{\prod _{i=t+1}^{N}\Gamma (s+m_i)\bigg \}x^{-2s},\, s=-m_1\bigg ]\\&\quad =x^{2m_1}\textrm{Res}\bigg [(\Gamma (u-m_1))^{N}(\Gamma (u))^t\bigg \{\prod _{i=t+1}^{N}\Gamma (u-m_1+m_i)\bigg \}x^{-2u},\, u=0\bigg ]\\&\quad \sim \big ( \Gamma (-m_1) \big )^N\frac{(-2\ln (x))^{t-1}}{(t-1)!} \bigg \{\prod ^N_{i=t+1} \Gamma \Big (m_i-m_1\Big )\bigg \} x^{2m_1}, \quad x\downarrow 0, \end{aligned}$$
  and all other residues are of lower order as $x\downarrow 0$. On arguing similarly to in part (i) we obtain the limiting form (2.21).
(iii) Parts (i) and (ii) imply that the PDF $f_Z(z)$ has a singularity at $z=0$ for all parameter values. Moreover, the PDF is bounded everywhere except for the singularity at the origin, and therefore the distribution of *Z* is unimodal with mode 0 for all parameter constellations.

$$\square $$

#### Tail Behaviour of the Distribution

##### Proposition 2.11

Suppose $m_1,\ldots ,m_N>-1/2$, $0\le |\beta _i|<\alpha _i$, $i=1,\ldots ,N.$ Denote the complementary CDF by $\bar{F}_Z(z)=\mathbb {P}(Z>z)$. Also, let $\mu _N=N^{-1}\sum _{i=1}^Nm_i$. Then

2.25 $$\begin{aligned} \bar{F}_Z(z)&\sim \frac{(2\pi )^\frac{N-1}{2}\eta }{\sqrt{N}}\bigg \{\prod _{i=1}^{N}\frac{\gamma _i^{2m_i+1}}{(2\alpha _i)^{m_i+1/2}}\bigg \}z^{\mu _N-\tfrac{1}{2N}}\nonumber \\&\quad \times \!\sum _{\sigma \in S_N^+}\!\bigg \{\prod _{k \in \sigma }\prod _{l \in \sigma ^c}(\lambda _k^+)^{\mu _N-m_k-\tfrac{N+1}{2N}}(\lambda _l^-)^{\mu _N-m_l-\tfrac{N+1}{2N}}\bigg \}\nonumber \\&\quad \times \textrm{e}^{-N\omega _\sigma ^{1/N}z^{1/N}}, \,\,\,z\rightarrow \infty , \end{aligned}$$

2.26 $$\begin{aligned} {F}_Z(z)&\sim \frac{(2\pi )^\frac{N-1}{2}\eta }{\sqrt{N}}\bigg \{\prod _{i=1}^{N}\frac{\gamma _i^{2m_i+1}}{(2\alpha _i)^{m_i+1/2}}\bigg \}(-z)^{\mu _N-\tfrac{1}{2N}}\nonumber \\&\quad \times \!\sum _{\sigma \in S_N^-}\!\bigg \{\prod _{k \in \sigma }\prod _{l \in \sigma ^c}(\lambda _k^+)^{\mu _N-m_k-\tfrac{N+1}{2N}}(\lambda _l^-)^{\mu _N-m_l-\tfrac{N+1}{2N}}\bigg \}\nonumber \\&\quad \times \textrm{e}^{-N\omega _\sigma ^{1/N}(-z)^{1/N}},\,\,\, z\rightarrow -\infty . \end{aligned}$$

##### Corollary 2.12

Suppose $$m_1,\ldots ,m_N>-1/2$$, $$0\le |\beta _i|<\alpha _i$$, $$i=1,\ldots ,N.$$ Then

2.27 $$\begin{aligned} f_Z(z)&\sim \frac{(2\pi )^\frac{N-1}{2}\eta }{\sqrt{N}}\bigg \{\prod _{i=1}^{N}\frac{\gamma _i^{2m_i+1}}{(2\alpha _i)^{m_i+1/2}}\bigg \}z^{\mu _N+\tfrac{1}{2N}-1}\nonumber \\&\quad \times \!\sum _{\sigma \in S_N^+}\!\bigg \{\prod _{k \in \sigma }\prod _{l \in \sigma ^c}(\lambda _k^+)^{\mu _N-m_k+\tfrac{1-N}{2N}}(\lambda _l^-)^{\mu _N-m_l+\tfrac{1-N}{2N}}\bigg \}\nonumber \\&\quad \times \textrm{e}^{-N\omega _\sigma ^{1/N}z^{1/N}},\,\,\, z\rightarrow \infty , \end{aligned}$$

2.28 $$\begin{aligned} f_Z(z)&\sim \frac{(2\pi )^\frac{N-1}{2}\eta }{\sqrt{N}}\bigg \{\prod _{i=1}^{N}\frac{\gamma _i^{2m_i+1}}{(2\alpha _i)^{m_i+1/2}}\bigg \}(-z)^{\mu _N+\tfrac{1}{2N}-1}\nonumber \\&\quad \times \!\sum _{\sigma \in S_N^-}\!\bigg \{\prod _{k \in \sigma }\prod _{l \in \sigma ^c}(\lambda _k^+)^{\mu _N-m_k+\tfrac{1-N}{2N}}(\lambda _l^-)^{\mu _N-m_l+\tfrac{1-N}{2N}}\bigg \}\nonumber \\&\quad \times \textrm{e}^{-N\omega _\sigma ^{1/N}(-z)^{1/N}},\,\,\, z\rightarrow -\infty . \end{aligned}$$

##### Remark 2.13

The limiting forms of Proposition 2.11 and Corollary 2.12 take a simpler form for certain parameter constellations. To illustrate this, we let $\omega _+=\min _{\sigma \in S_N^+} \omega _\sigma$, and suppose that this minimum is attained for just a single $\sigma \in S_N^+$. We then have that $\exp (-N\omega _\sigma ^{1/N}z^{1/N})\ll \exp (-N\omega _+^{1/N}z^{1/N})$, as $z\rightarrow \infty$, for all $\sigma \in S_N^+$ except for the one at which the minimum value $w_+$ is attained. In this case, the limiting form (2.26) reduces to

$$\begin{aligned} \bar{F}_Z(z)\sim \frac{(2\pi )^\frac{N-1}{2}\eta }{\sqrt{N}}\bigg \{\prod _{i=1}^{N}\frac{\gamma _i^{2m_i+1}}{(2\alpha _i)^{m_i+1/2}}\bigg \}z^{\mu _N-\tfrac{1}{2N}}\textrm{e}^{-N\omega _+^{1/N}z^{1/N}},\quad z\rightarrow \infty . \end{aligned}$$

The limiting forms of the PDF and CDF of *Z* also take a simpler form when $\beta _1=\cdots =\beta _N=0$. In this case,

2.29 $$\begin{aligned} f_Z(z)&\sim \frac{\pi ^{(N-1)/2}\xi \eta }{2^{N(\mu _N-1)+3/2}\sqrt{N}}(\xi |z|)^{\mu _N+\frac{1}{2N}-1}\textrm{e}^{-N(\xi |z|)^{1/N}}, \quad |z|\rightarrow \infty , \end{aligned}$$

2.30 $$\begin{aligned} \bar{F}_Z(z)&\sim \frac{\pi ^{(N-1)/2}\eta }{2^{N(\mu _N-1)+3/2}\sqrt{N}}(\xi z)^{\mu _N-\frac{1}{2N}}\textrm{e}^{-N(\xi z)^{1/N}}, \quad z\rightarrow \infty , \end{aligned}$$

2.31 $$\begin{aligned} F_Z(z)&\sim \frac{\pi ^{(N-1)/2}\eta }{2^{N(\mu _N-1)+3/2}\sqrt{N}}(-\xi z)^{\mu _N-\frac{1}{2N}}\textrm{e}^{-N(-\xi z)^{1/N}}, \quad z\rightarrow -\infty . \end{aligned}$$

These limiting forms can also be easily derived through the following alternative approach. The limiting form (2.30) follows immediately on applying the limiting form (A.8) to the formula (2.2) for the PDF of *Z* in the case $\beta _1=\cdots =\beta _N=0$. To obtain (2.31) we apply the limiting form (2.30) to the formula $\bar{F}_Z(z)=\int _z^\infty f_Z(x) \,\textrm{d}x$, and (2.31) then follows since, for $a\in \mathbb {R}$ and $b,r>0$, we have that $\int _z^\infty x^a\textrm{e}^{-bx^r}\,\textrm{d}x\sim (br)^{-1}z^{a-r+1}\textrm{e}^{-bz^r}$, as $z\rightarrow \infty$, which is easily proved by a standard integration by parts argument. The limiting form (2.32) follows immediately from (2.31) since the distribution of *Z* is symmetric about the origin in the $\beta _1=\cdots =\beta _N=0$ case.

We will make use of Lemma 2.1 of [22] in proving Proposition 2.11.

##### Lemma 2.14

(Arendarczyk and Dȩbicki [22]). Let $X_1$ and $X_2$ be independent, non-negative random variables such that $\bar{F}_{X_i}(x)\sim A_ix^{r_i}\exp (-b_ix^{a_i})$ as $x\rightarrow \infty$, for $i=1,2$. Then

$$\begin{aligned} \bar{F}_{X_1X_2}(x)\sim Ax^{r}\exp (-bx^{a}), \quad x\rightarrow \infty , \end{aligned}$$

where, for $$c:=a_1+a_2$$,

$$\begin{aligned} a&=\frac{a_1a_2}{c},\quad b=b_1^{a_2/c}b_2^{a_1/c}\bigg (\Big (\frac{a_1}{a_2}\Big )^{a_2/c}+\Big (\frac{a_2}{a_1}\Big )^{a_1/c}\bigg ), \quad r=\frac{a_1a_2+2a_1r_2+2a_2r_1}{2c}, \\ A&=\frac{\sqrt{2\pi }A_1A_2}{\sqrt{c}}(a_1b_1)^{\frac{a_2-2r_1+2r_2}{2c}}(a_2b_2)^{\frac{a_1-2r_2+2r_1}{2c}}.\end{aligned}$$

##### Proof of Proposition 2.11

Let $Z_\sigma ^+=\prod _{k \in \sigma }\prod _{l \in \sigma ^c}X_k^-X_l^+$, $\sigma \in S_N^+$, and $Z_\sigma ^-=\prod _{k \in \sigma }\prod _{l \in \sigma ^c}X_k^-X_l^+$, $\sigma \in S_N^-$. We can then derive the following limiting form by induction on *N*: As $z\rightarrow \infty$,

$$\begin{aligned} \bar{F}_{Z_\sigma ^+}(z)&\sim \frac{(2\pi )^\frac{N-1}{2}}{\sqrt{N}}\bigg \{\prod _{i=1}^{N}\frac{\gamma _i^{2m_i+1}}{(2\alpha _i)^{m_i+1/2}\Gamma (m_i+1/2)}\bigg \}z^{\mu _N-\tfrac{1}{2N}}\nonumber \\&\quad \times \bigg \{\prod _{k \in \sigma }\prod _{l \in \sigma ^c}(\lambda _k^+)^{\mu _N-m_k-\tfrac{N+1}{2N}}(\lambda _l^-)^{\mu _N-m_l-\tfrac{N+1}{2N}}\bigg \}\textrm{e}^{-N\omega _\sigma ^{1/N}z^{1/N}}, \quad \sigma \in S_N^+. \end{aligned}$$

The base case $N=2$ is given in the proof of Proposition 2.11 of [8] and the general case follows by induction on *N* using Lemma 2.14. Similarly, we have that, as $z\rightarrow -\infty$,

$$\begin{aligned} {F}_{Z_\sigma ^-}(z)&\sim \frac{(2\pi )^\frac{N-1}{2}}{\sqrt{N}}\bigg \{\prod _{i=1}^{N}\frac{\gamma _i^{2m_i+1}}{(2\alpha _i)^{m_i+1/2}\Gamma (m_i+1/2)}\bigg \}(-z)^{\mu _N-\tfrac{1}{2N}}\\&\quad \times \bigg \{\prod _{k \in \sigma }\prod _{l \in \sigma ^c}(\lambda _k^+)^{\mu _N-m_k-\tfrac{N+1}{2N}}(\lambda _l^-)^{\mu _N-m_l-\tfrac{N+1}{2N}}\bigg \}\nonumber \\&\quad \times \textrm{e}^{-N\omega _\sigma ^{1/N}(-z)^{1/N}}, \quad \sigma \in S_N^-. \end{aligned}$$

The limiting forms (2.26) and (2.27) are now obtained from the formulas $\bar{F}_{Z}(z)=\sum _{\sigma \in S_N^+ }\bar{F}_{Z^+_\sigma }(z)$ and ${F}_{Z}(z)=\sum _{\sigma \in S_N^-}F_{Z_\sigma ^-}(z)$, respectively. $\square$

##### Proof of Corollary 2.12

We obtain the limiting forms (2.28) and (2.29) from the limiting forms (2.26) and (2.27), respectively, by using the relations $f_Z(z)=-\bar{F}_Z'(z)$ and $f_Z(z)=F_Z'(z)$ and retaining the leading order terms.

$$\square $$

#### Asymptotic Behaviour of the Quantile Function

##### Proposition 2.15

For $0<p<1$, write $Q(p)=F_Z^{-1}(p)$ for the quantile function of $Z=\prod _{i=1}^{N}X_i$. Let $m_1,\ldots ,m_N>-1/2$, $0\le |\beta _i|<\alpha _i$, $i=1,\ldots ,N$. Denote $\omega _+=\min _{\sigma \in S_N^+} \omega _\sigma$ and $\omega _-=\min _{\sigma \in S_N^-} \omega _\sigma$. Then

2.32 $$\begin{aligned} Q(p)&\sim \frac{1}{N^N\omega _+}\big (-\ln (1-p)\big )^N,\quad p\rightarrow 1, \end{aligned}$$

2.33 $$\begin{aligned} Q(p)&\sim -\frac{1}{N^N\omega _-}\big (-\ln (p)\big )^N,\quad p\rightarrow 0. \end{aligned}$$

We begin by proving the following lemma.

##### Lemma 2.16

Let $a,A,N,z>0$ and $r\in \mathbb {R}$. Let $g:(0,\infty )\rightarrow \mathbb {R}$ be a function such that $g(x)\rightarrow 0$ as $x\rightarrow \infty$. Consider the equation

2.34 $$\begin{aligned} Ax^r\exp (-ax^{1/N})(1+g(x))=z, \end{aligned}$$

and notice that there is a unique solution *x* provided *z* is sufficiently small. Then, as $z\rightarrow 0$,

2.35 $$\begin{aligned} x\sim \frac{1}{a^N}\big (-\ln (z)\big )^N. \end{aligned}$$

##### Proof

From equation ($2.35$) we get that $x= (\ln (w)+r\ln (x)+\ln (h(x)))^N/a^N$, where $w=A/z$ and $h(x)=1+g(x)$. From this equation it is clear that $x=O((\ln (w))^N)$ as $w\rightarrow \infty$. From this, we deduce that $\ln (x)=O(\ln (\ln (w))$ as $w\rightarrow \infty$, and that $\ln (h(x))\rightarrow 0$ as $w\rightarrow \infty$ (because $\ln (1+u)\rightarrow 0$ as $u\rightarrow 0$). Thus, $x\sim (\ln (w))^N/{a^N}\sim (-\ln (z))^N/{a^N}$ as $z\rightarrow 0$. $\square$

##### Proof of Proposition 2.15

We derive the limiting form (2.33); the derivation of (2.34) is similar and thus omitted. Since *Q*(*p*) satisfies $\bar{F}_Z(Q(p))=1-p$, it can be seen upon applying the limiting form (2.26) that *Q*(*p*) solves an equation of the form (2.35) with $z=1-p$ and $a=N\omega _+^{1/N}$. The limiting form (2.33) thus follows upon applying (2.36) with these values of *z* and *a*. $\square$

## Special Cases

### Product of *N* Independent Asymmetric Laplace Random Variables

In this section, we apply the general formulas of Section 2 to obtain closed-form formulas for the PDF, CDF and characteristic function of the product of *N* independent asymmetric Laplace random variables. We recall that the $\textrm{VG}(1/2,\alpha ,\beta ,0)$ distribution corresponds to the asymmetric Laplace distribution (with zero location parameter), denoted by $\textrm{AL}(\alpha ,\beta )$, with density $f(x)=(2\alpha )^{-1}(\alpha ^2-\beta ^2)\textrm{e}^{\beta x-\alpha |x|}$, $x\in \mathbb {R}$ (see [2]). Further setting $\beta =0$ yields the classical Laplace distribution, denoted by $\textrm{Laplace}(\alpha )$, with density $f(x)=(\alpha /2)\textrm{e}^{-\alpha |x|}$, $x\in \mathbb {R}$.

#### Corollary 3.1

Let $X_i\sim \textrm{AL}(\alpha _i,\beta _i)$, $i=1,\ldots ,N$ be independent asymmetric Laplace random variables, with $0\le |\beta _i|<\alpha _i$, $i=1,\ldots ,N$. Let $Z=\prod _{i=1}^{N}X_i$. Then

(i) For $$z\in \mathbb {R}$$,
  $$\begin{aligned} f_Z(z)=&\frac{1}{2^N\xi } \bigg \{\prod _{i=1}^{N}\gamma _i^2\bigg \} \sum _{\sigma \in A(z)}G^{N,0}_{0,N} \bigg (\omega _\sigma |z| \,\bigg |\, \begin{aligned} -\atop 0,\ldots ,0 \end{aligned} \bigg ). \end{aligned}$$
(ii) We have
  $$\begin{aligned} F_Z(z)&=1-\frac{1}{2^N\xi } \bigg \{\prod _{i=1}^{N}\gamma _i^2\bigg \} \sum _{\sigma \in S_N^+}\frac{1}{\omega _\sigma }\bigg \{1-G^{N,1}_{1,N+1} \bigg ( \omega _\sigma z\,\bigg |\, \begin{aligned}&1 \\ 1, \ldots&, 1,0 \end{aligned} \bigg )\bigg \}, \ z>0, \\ F_Z(z)&=\frac{1}{2^N\xi } \bigg \{\prod _{i=1}^{N}\gamma _i^2\bigg \}\sum _{\sigma \in S_N^-}\frac{1}{\omega _\sigma }\bigg \{1-G^{N,1}_{1,N+1} \bigg ( -\omega _\sigma z\,\bigg |\, \begin{aligned}&1 \\ 1, \ldots&, 1,0 \end{aligned} \bigg )\bigg \}, \quad z<0. \end{aligned}$$
(iii) For $$t\in \mathbb {R}$$,
  2.36 $$\begin{aligned} \varphi _Z(t)&=\frac{\textrm{i}}{2^N\xi t}\bigg \{\prod _{j=1}^{N}\gamma _j^2\bigg \}\bigg \{\sum _{\sigma \in S_N^+}G^{N,1}_{1,N} \bigg (\frac{\textrm{i}\omega _\sigma }{t}\, \bigg |\, \begin{aligned} 0 \atop 0, \ldots ,0 \end{aligned} \bigg )\nonumber \\&\quad -\sum _{\sigma \in S_N^-}G^{N,1}_{1,N} \bigg (-\frac{\textrm{i}\omega _\sigma }{t}\, \bigg |\, \begin{aligned} 0 \atop 0, \ldots ,0 \end{aligned} \bigg )\bigg \}. \end{aligned}$$

#### Corollary 3.2

Let $X_i\sim \textrm{Laplace}(\alpha _i)$, $i=1,\ldots ,N$, be independent Laplace random variables. Let $Z=\prod _{i=1}^{N}X_i$. Then the PDF, CDF and characteristic function of *Z* are given by

$$\begin{aligned} f_Z(z)&= \frac{\xi }{2}G^{N,0}_{0,N} \bigg (\xi |z|\, \bigg |\, \begin{aligned} -\atop 0,\ldots ,0 \end{aligned} \bigg ),\quad z\in \mathbb {R}, \\ F_Z(z)&=\frac{1}{2}+\frac{\textrm{sgn}(z)}{2}G^{N,1}_{1,N+1}\bigg ( \xi |z|\, \bigg |\,{ 1\atop 1,\ldots ,1,0}\bigg ),\quad z\in \mathbb {R},\\ \varphi _Z(t)&=\frac{\xi }{2^{N-1}\pi ^{(N-1)/2}}|t|^{-1}G^{2N-1,1}_{1,2N-1} \bigg ( \frac{\xi ^2}{4^{N-1}t^2} \,\bigg |\, \begin{aligned} \frac{1}{2}\atop 0, \ldots ,0, \,\frac{1}{2},\ldots , \frac{1}{2} \end{aligned} \bigg ),\quad t\in \mathbb {R}. \end{aligned}$$

#### Remark 3.3

In the case $N=2$, we can apply the reduction formula (A.5) to formula (3.1) to obtain the following expression for the characteristic function of $Z=X_1X_2$, where $X_1\sim \textrm{AL}(\alpha _1,\beta _1)$ and $X_2\sim \textrm{AL}(\alpha _2,\beta _2)$ are independent. For $t\in \mathbb {R}$,

3.1 $$\begin{aligned} \varphi _Z(t)&=\frac{\gamma _1^2\gamma _2^2}{4\alpha _1\alpha _2}\bigg (\frac{\textrm{i}}{t}\bigg )^{1/2}\bigg \{\frac{\textrm{e}^{\textrm{i}\lambda _1^-\lambda _2^-/(2t)}}{(\lambda _1^-\lambda _2^-)^{1/2}}W_{-\frac{1}{2},0}\bigg (\frac{\textrm{i}\lambda _1^-\lambda _2^-}{t}\bigg )+\frac{\textrm{e}^{\textrm{i}\lambda _1^+\lambda _2^+/(2t)}}{(\lambda _1^+\lambda _2^+)^{1/2}}\nonumber \\&\quad \times W_{-\frac{1}{2},0}\bigg (\frac{\textrm{i}\lambda _1^+\lambda _2^+}{t}\bigg )+\frac{\textrm{e}^{-\textrm{i}\lambda _1^-\lambda _2^+/(2t)}}{(\lambda _1^-\lambda _2^+)^{1/2}}W_{-\frac{1}{2},0}\bigg (-\frac{\textrm{i}\lambda _1^-\lambda _2^+}{t}\bigg )\nonumber \\&\quad +\frac{\textrm{e}^{-\textrm{i}\lambda _1^+\lambda _2^-/(2t)}}{(\lambda _1^+\lambda _2^-)^{1/2}}W_{-\frac{1}{2},0}\bigg (-\frac{\textrm{i}\lambda _1^+\lambda _2^-}{t}\bigg )\bigg \}, \nonumber \end{aligned}$$

where $W_{\kappa ,\mu }(x)$ is a Whittaker function (see [19, equation 13.14.3] for a definition). This formula corrects the formula of part (iii) of Corollary 3.1 of [8] in which the denominator in the argument of the Whittaker functions was erroneously given by 2*t* rather than by *t*.

#### Proof of Corollary 3.1

For parts (i) and (iii), we simply set $m_1=\cdots =m_N=1/2$ in (2.7) and (2.19), respectively. The proof of part (ii) is more involved. We prove this part for $z>0$; the case $z<0$ is similar and thus omitted. For $z>0$, we integrate the PDF from part (i) to get

3.2 $$\begin{aligned} F_Z(z)&=1-\frac{1}{2^N\xi } \bigg \{\prod _{i=1}^{N}\gamma _i^2\bigg \} \sum _{\sigma \in A(z)}\int _z^\infty G^{N,0}_{0,N} \bigg (\omega _\sigma y \,\bigg |\, \begin{aligned} -\atop 0,\ldots ,0 \end{aligned} \bigg ) \,\textrm{d}y \nonumber \\&=1-\frac{1}{2^N\xi } \bigg \{\prod _{i=1}^{N}\gamma _i^2\bigg \} \sum _{\sigma \in A(z)}\frac{1}{\omega _\sigma }\int _{\omega _\sigma z}^\infty G^{N,0}_{0,N} \bigg (u \,\bigg |\, \begin{aligned} -\atop 0,\ldots ,0 \end{aligned} \bigg ) \,\textrm{d}u \nonumber \\&=1-\frac{1}{2^N\xi } \bigg \{\prod _{i=1}^{N}\gamma _i^2\bigg \} \sum _{\sigma \in A(z)}\frac{1}{\omega _\sigma } \bigg [G^{N,1}_{1,N+1} \bigg (u \,\bigg |\, \begin{aligned} 1\atop 1,\ldots ,1,0 \end{aligned} \bigg ) \bigg ]_{\omega _\sigma z}^\infty , \end{aligned}$$

where we used the integral formula (A.7) in the last step. Finally, we calculate the limit

3.3 $$\begin{aligned} \lim _{u\rightarrow \infty }G^{N,1}_{1,N+1} \bigg (u \,\bigg |\, \begin{aligned} 1\atop 1,\ldots ,1,0 \end{aligned} \bigg )=\lim _{v\rightarrow 0}G^{1,N}_{N+1,1} \bigg (v \,\bigg |\, \begin{aligned} 0,\ldots ,0,1\atop 0 \end{aligned} \bigg )=1, \end{aligned}$$

where we used the identity (A.4) (and set $v=1/u$) to obtain the equality, and the final limit is easily computed by writing the Meijer *G*-function as a contour integral using (A.1) and then computing the residues as we did in the proof of Proposition 2.10; we omit the details. Applying the limit (3.3) to equation (3.2) now yields the desired formula for $F_{Z}(z)$ for $z>0$. $\square$

#### Proof of Corollary 3.2

For the PDF, set $\beta _1=\cdots =\beta _N=0$ in part (i) of Corollary 3.1. For the CDF and characteristic function, set $m_1=\cdots =m_N=1/2$ in formulas (2.13) and (2.18), respectively. $\square$

### Product of Independent Zero Mean Normal and Laplace Random Variables

Recall that the $\textrm{VG}(1/2,\alpha ,0,0)$ distribution corresponds to a Laplace distribution with density $f(x)=(\alpha /2)\textrm{e}^{-\alpha |x|}$, $x\in \mathbb {R}$. The product of independent zero mean normal random variables is also VG distributed: Let $X_1\sim N(0,\sigma _1^2)$ and $X_2\sim N(0,\sigma _2^2)$ be independent. Then, by [4, Proposition 1.2], $X_1X_2\sim \textrm{VG}(0,1/(\sigma _1\sigma _2),0,0)$.

#### Corollary 3.4

Let $X_1,\ldots ,X_{2M}$ and $Y_1,\ldots ,Y_N$ be mutually independent random variables with $X_i\sim N(0,\sigma _i^2)$, $i=1,\ldots ,2M$, and $Y_j\sim \textrm{Laplace}(\alpha _j)$, $j=1,\ldots ,N$. Let $Z=(\prod _{i=1}^{2M}X_i)(\prod _{j=1}^N Y_j)$. Denote $\nu =(\prod _{i=1}^{2M}\sigma _i^{-1})(\prod _{j=1}^N\alpha _j)$. Also, let $\textbf{0}$ and $\textbf{0}'$ be the zero vectors of dimensions $2M+N$ and $2M+N-1$, respectively, and let $\varvec{\frac{1}{2}}$ be a vector of dimension *N* with all entries set to 1/2. Then the PDF, CDF and characteristic function of *Z* are given by

$$\begin{aligned} f_Z(z)&=\frac{\nu }{2^{M+N} \pi ^{M+N/2}} G^{2(M+N),0}_{0,2(M+N)} \bigg ( \frac{\nu ^{2}z^2}{4^{M+N}}\, \bigg |\, \begin{aligned} - \atop \textbf{0}, \varvec{\frac{1}{2}} \end{aligned} \bigg ),\quad z\in \mathbb {R}, \\ F_Z(z)&=\frac{1}{2}+ \frac{\nu z }{ 2^{M+N+1} \pi ^{M+N/2} } G^{2(M+N),1}_{1,2(M+N)+1}\bigg ( \frac{\nu ^2z^2}{4^{M+N}} \,\bigg |\,{ \frac{1}{2} \atop \textbf{0}, \varvec{\frac{1}{2}},-\frac{1}{2}}\bigg ),\quad z\in \mathbb {R},\\ \varphi _Z(t)&=\frac{\nu |t|^{-1}}{2^{M+N-1}\pi ^{M+(N-1)/2}}G^{2(M+N)-1,1}_{1,2(M+N)-1} \bigg ( \frac{\nu ^2}{4^{M+N-1}t^2} \,\bigg |\, \begin{aligned} \tfrac{1}{2}\atop \textbf{0}', \varvec{\frac{1}{2}} \end{aligned} \bigg ),\quad t\in \mathbb {R}. \end{aligned}$$

#### Proof

To obtain the formulas for the PDF, CDF and characteristic function, we apply formulas (2.2), (2.10) and (2.18), respectively, with $m_1=\cdots =m_M=0$, $m_{M+1}=\cdots =m_{M+N}=1/2$ and $\xi =\nu$. $\square$

### Product of Correlated Zero Mean Normal Random Variables

Consider a 2*N*-dimensional multivariate normal random vector $(X_1,\ldots ,X_{2N})^\intercal$ with zero mean vector and block diagonal covariance matrix $\Sigma =\textrm{diag}(\Sigma _1,\Sigma _2,\ldots ,\Sigma _N)$, where $\Sigma _i$ is a $2\times 2$ matrix given by

$$\begin{aligned} \Sigma _i=\left[ \begin{aligned}&\quad \,\sigma _{2i-1}^2&\rho _i \sigma _{2i-1} \sigma _{2i} \\  &\rho _i \sigma _{2i-1} \sigma _{2i}&\sigma _{2i}^2 \quad \,\,\, \end{aligned} \right] , \quad i=1,\ldots ,N, \end{aligned}$$

and $\rho _1,\ldots ,\rho _N\in (-1,1)$ are the correlation coefficients $\rho _i=\textrm{Cov}(X_{2i-1},X_{2i})/(\sigma _{2i-1} \sigma _{2i})$. In this section, we apply Theorem 2.1 to write down an exact formula for the PDF of the product $Z=\prod _{i=1}^{2N}X_i$. To this end, we note that the product $X_{2i-1}X_{2i}$ has a VG distribution:

3.4 $$\begin{aligned} X_{2i-1}X_{2i}\sim \textrm{VG}\bigg (0,\frac{1}{\sigma _{2i-1}\sigma _{2i}(1-\rho _i^2)},\frac{\rho _i}{\sigma _{2i-1}\sigma _{2i}(1-\rho _i^2)},0\bigg ), \quad i=1,\ldots ,N \end{aligned}$$

(see [18, Theorem 1]). We remark that starting with the work of [23, 24], the distribution of the product of two correlated zero mean normal random variables has received considerable attention in the statistics literature (for an overview see [25, 26]). On combining formula (2.1) for the VG product PDF with (3.4), we obtain the following formula for the PDF of $Z=\prod _{i=1}^{2N}X_i$.

#### Corollary 3.5

Let the above notations hold, and set $s=\prod _{i=1}^{2N}\sigma _i$ and $\tau =\prod _{i=1}^N(1-\rho _i^2)$. Then

3.5 $$\begin{aligned} f_Z(z)&= \frac{1}{2^{2N-1}\pi ^{N}s\sqrt{\tau }} \sum ^\infty _{j_1=0} \ldots \sum ^\infty _{j_N=0} \frac{(2\rho _{1})^{j_1}}{ j_1! } \ldots \frac{(2\rho _N)^{j_N} }{j_N!} a_{j_1,\ldots ,j_N}(z) \nonumber \\  &\quad \times G^{2N,0}_{0,2N} \bigg ( \frac{z^{2}}{4^Ns^2\tau ^2}\, \bigg |\, { - \atop \tfrac{j_1}{2},\ldots , \tfrac{j_N}{2}, \tfrac{j_1}{2}, \ldots , \tfrac{j_N}{2} } \bigg ), \quad z\in \mathbb {R}. \end{aligned}$$

#### Remark 3.6

Note that the PDF (3.5) simplifies to a single Meijer *G*-function if $\rho _1=\cdots =\rho _N=0$, in which case we recover the formula of [15] for the product of 2*N* independent zero mean normal random variables. Similarly, if $N-k$ of the correlation coefficients are equal to zero then the PDF (3.5) reduces to a *k*-fold infinite series.

There is interest in developing distributional theory for the product of three or more correlated zero mean normal random variables (see the review [27] for an overview); however, to date few explicit formulas are known for these distributions. Formula (3.5) is one of the first such explicit formulas, treating the case of a block diagonal covariance matrix, and we hope the result inspires further research into the study of the exact distribution of the product of more than two correlated zero mean normal random variables.

## Data Availability

This manuscript has no associated data.

## The Meijer *G*-function

Here, we define the Meijer *G*-function, and present some of its relevant basic properties, all of which can be found in [19, 21].

The *Meijer G-function* is defined, for $x\in \mathbb {R}$, by the contour integral

A.1 $$\begin{aligned} G^{m,n}_{p,q}\bigg (x \, \bigg |\, {a_1,\ldots , a_p \atop b_1,\ldots ,b_q} \bigg )=\frac{1}{2\pi \textrm{i}}\int _L\frac{\prod _{j=1}^m\Gamma (b_j-s)\prod _{j=1}^n\Gamma (1-a_j+s)}{\prod _{j=n+1}^p\Gamma (a_j-s)\prod _{j=m+1}^q\Gamma (1-b_j+s)}x^s\,\textrm{d}s, \end{aligned}$$

where the path *L* separates the poles of the gamma functions $\Gamma (b_j-s)$ from the poles of the gamma functions $\Gamma (1-a_j+s)$, and we employ the convention that the empty product is 1.

The Meijer *G*-function satisfies the identities

A.2 $$\begin{aligned} G_{p,q}^{m,n}\bigg (x \; \bigg | \;{a_1,\ldots ,a_{p-1},b_1 \atop b_1,\ldots ,b_q}\bigg )&=G_{p-1,q-1}^{m-1,n}\bigg (x \; \bigg | \;{a_1,\ldots ,a_{p-1} \atop b_2,\ldots ,b_q}\bigg ), \quad m,p,q\ge 1, \end{aligned}$$

A.3 $$\begin{aligned} x^\alpha G_{p,q}^{m,n}\bigg (x \; \bigg | \;{a_1,\ldots ,a_p \atop b_1,\ldots ,b_q}\bigg )&=G_{p,q}^{m,n}\bigg (x \, \bigg | \,{a_1+\alpha ,\ldots ,a_p+\alpha \atop b_1+\alpha ,\ldots ,b_q+\alpha }\bigg ), \end{aligned}$$

A.4 $$\begin{aligned} G^{m,n}_{p,q}\bigg (x \; \bigg |\; {a_1,\ldots , a_p \atop b_1,\ldots ,b_q} \bigg )&=G^{n,m}_{q,p}\bigg (x^{-1} \; \bigg |\; {1-b_1,\ldots , 1-b_q \atop 1-a_1,\ldots ,1-a_p} \bigg ). \end{aligned}$$

The Whittaker function arises as a special case of the Meijer *G*-function:

A.5 $$\begin{aligned} G^{2,1}_{1,2} \bigg ( x\, \bigg |\, { a \atop b,c } \bigg )&=\Gamma (b-a+1)\Gamma (c-a+1)x^{(b+c-1)/2}\textrm{e}^{x/2}W_{\frac{1}{2}(2a-b-c-1),\frac{1}{2}(b-c)}(x). \end{aligned}$$

On combining equations 5.4(1) and 5.4(13) of [21], we obtain the indefinite integral formula

A.6 $$\begin{aligned} \int x^{\alpha -1}G_{p,q}^{m,n}\bigg (x \; \bigg | \;{a_1,\ldots ,a_p \atop b_1,\ldots ,b_q}\bigg )\,\textrm{d}x=x^\alpha G_{p+1,q+1}^{m,n+1}\bigg (x \, \bigg | \,{1-\alpha ,a_1,\ldots ,a_p \atop b_1,\ldots ,b_q,-\alpha }\bigg ). \end{aligned}$$

Setting $\alpha =1$ in (A.6) and applying (A.3) yields

A.7 $$\begin{aligned}&\int G_{p,q}^{m,n}\bigg (x \; \bigg | \;{a_1,\ldots ,a_p \atop b_1,\ldots ,b_q}\bigg )\,\textrm{d}x\nonumber \\&\quad = G_{p+1,q+1}^{m,n+1}\bigg (x \, \bigg | \,{1,a_1+1,\ldots ,a_p+1 \atop b_1+1,\ldots ,b_m+1,0,b_{m+1}+1,\ldots , b_q+1}\bigg ). \end{aligned}$$

The following limiting form is given in [21, Section 5.7, Theorem 5]. For $x>0$,

A.8 $$\begin{aligned} G^{q,0}_{p,q}\bigg (x \; \bigg |\; {a_1,\ldots , a_p \atop b_1,\ldots ,b_q} \bigg )\sim \frac{(2\pi )^{(\sigma -1)/2}}{\sigma ^{1/2}}x^\theta \exp \big (-\sigma x^{1/\sigma }\big ), \quad \text { as } x\rightarrow \infty , \end{aligned}$$

where $$\sigma =q-p$$ and $$\theta =\sigma ^{-1}\{(1-\sigma )/2+\sum _{i=1}^qb_i-\sum _{i=1}^pa_i\}$$.
